# Mapping Peak Expiratory Flow in Healthy Pakistani Adults: Insights from Wah Cantt

**DOI:** 10.4314/ejhs.v35i4.3

**Published:** 2025-07

**Authors:** Aimen Iqbal, Waleed Nasir, Faizan Akram, Robina Mushtaq, Tooba Iqbal, Fatima Nasir

**Affiliations:** 1 Wah Medical College, National University of Medical Sciences PK; 2 Riphah International hospital, Sihala campus, PK

**Keywords:** Peak Expiratory Flow Rate, Lung Function, Spirometry, Pulmonary Assessment, Demographic Variation

## Abstract

**Background:**

Respiratory assessments frequently utilize Peak Expiratory Flow Rate (PEFR) as a fundamental measure of lung function. However, standard reference ranges may not accurately represent variations across different demographic groups. This study aims to address this gap by examining the variability of PEFR among healthy Pakistani adults.

**Methods:**

This cross-sectional study was conducted over a six-month period at a tertiary care hospital in Wah Cantt, Pakistan, involving 400 consenting participants. A consecutive sampling technique was employed. Anthropometric measurements, including height and weight, were obtained using a stadiometer and calibrated weighing scale, respectively. Each participant performed three PEFR maneuvers using a peak flow meter, and the highest value was recorded. Additional data, including age and gender, were collected using a structured proforma. Data was analyzed using SPSS.

**Results:**

PEFR showed a strong positive correlation with height (0.616 in males 0.767 in females) and a negative correlation with age (−0.418 in males and −0.115 in females). Male participants exhibited significantly higher PEFR values compared to females.

**Conclusion:**

The findings indicate that PEFR is influenced by gender, height, and age. Specifically, males tend to have higher PEFR values; PEFR increases with greater height and decreases with advancing age. These results underscore the importance of considering demographic-specific factors when interpreting PEFR values and support the development of localized reference standards for clinical use.

## Introduction

Peak Expiratory Flow Rate (PEFR) is a vital parameter in assessing lung function and serves as a fundamental tool in the diagnosis and management of respiratory disorders. Requiring minimal patient training, PEFR offers clinicians valuable insights into an individual's pulmonary health and their response to therapeutic interventions. Its ease of use, affordability, and the widespread availability of peak flow meters contribute to its reliability and practicality in both acute and general clinical settings (1).

PEFR is most utilized in the management of asthma. In the short term, PEFR monitoring aids in managing acute exacerbations, identifying triggers, and assessing treatment efficacy. Over the long term, it supports tracking disease control and anticipating potential flare-ups (2). The British Thoracic Society (BTS) guidelines offer a standardized framework for classifying asthma exacerbations as mild, moderate, or severe (3). For chronic obstructive pulmonary disease (COPD), a lifelong and often debilitating condition, PEFR can serve as a valuable predictor of episodic instability and acute symptom worsening (4,5).

Beyond asthma and COPD, PEFR has demonstrated utility in a variety of clinical and public health contexts. Recent studies have linked reduced PEFR to an increased risk of pneumonia in the elderly (6). Additionally, PEFR has shown promise in evaluating the nutritional status of frail older adults, highlighting its potential as a simple yet powerful diagnostic tool (7). In occupational health, PEFR is useful in assessing the respiratory impact of workplace exposures and can be incorporated into pre-employment medical screenings to establish baseline lung function (8,9).

Despite its clinical reliability, PEFR values are influenced by various factors, including age, sex, height, weight, and ethnicity. Recent research indicates that PEFR demonstrates lower inter-subject variability across age groups than previously reported, and predictive models incorporating demographic variables further strengthen its clinical utility (10,11).

In Pakistan, while PEFR has been studied in subgroups such as medical students and industrial workers, there is a notable lack of data on healthy adult populations (12,13,14). This gap is particularly concerning, given the significant burden of asthma and COPD in the country—conditions that account for nearly one-quarter of primary healthcare visits (15). Encouragingly, research by Khan et al. demonstrated the feasibility and benefits of integrated asthma-COPD care at public health facilities, even in low-resource settings (16).

This study seeks to address this data gap by assessing PEFR values among a diverse cohort of healthy adults in Wah Cantt. The study further explores how variables such as age, gender, and anthropometric characteristics affect PEFR. Ultimately, the goal is to inform targeted healthcare strategies and promote community-based respiratory interventions that reflect the demographic realities of the population.

## Materials and Methods

**Study design**: A cross-sectional observational study was conducted at a tertiary care hospital in Wah Cantt over a six-month period. The study was designed as a single-center, hospital-based investigation to evaluate Peak Expiratory Flow Rate (PEFR) in healthy adults.

**Sample size and sampling technique**: Using the WHO sample size calculator, a sample size of 385 was determined based on a 95% confidence interval, 50% population proportion, and a 5% margin of error. A total of 400 participants, aged 18 to 65 years, were selected using a consecutive sampling method. Participants were screened to ensure they met eligibility criteria and could perform the PEFR maneuver accurately.

**Inclusion and exclusion criteria**: Inclusion criteria included healthy adults between 18 and 65 years of age who could understand and perform the PEFR test. Exclusion criteria comprised individuals with known conditions that could impair PEFR values, such as acute or chronic respiratory illness, a history of smoking, morbid obesity, structural thoracic abnormalities, and pregnancy. Participants experiencing respiratory distress or shortness of breath at the time of assessment were also excluded.

**Data collection procedure**: Written informed consent was obtained from all participants. A structured questionnaire was used to gather demographic information. Height and weight were measured using a stadiometer and calibrated weighing scale to calculate Body Mass Index (BMI). PEFR was measured using a peak flow meter (Peak Flow Meter (Adult) PF120A, ROSSMAX brand) following standard procedure, with the highest value from three attempts recorded for analysis.

**Data analysis**: Data was analyzed using SPSS version 23. Descriptive statistics were used to summarize both qualitative and quantitative variables. Gender was reported as frequencies and percentages, while variables such as age, height, weight, BMI, and PEFR were expressed as means with standard deviations. Independent t-tests and ANOVA were used to examine relationships between PEFR and anthropometric variables. A p-value of <0.05 was considered statistically significant.

**Ethical considerations**: Ethical approval was obtained from the Ethical Research Committee of Wah Medical College (Letter No. WMC/ERC/IRB/043). Written informed consent was obtained from all participants, who were informed of their right to withdraw from the study at any time. Confidentiality and anonymity of participant data were maintained throughout the study.

## Results

The study included 400 participants of Pakistani Asian descent, with a mean age of 35.06 years (males: 35.39 years; females: 33.5 years). The gender distribution was 66.7% male and 33.3% female. Table 1 presents the mean height and PEFR values by age and gender, along with corresponding standard deviations.

**Table 1:** Mean values and standard deviation of Height (cm) and PEFR by age and gender

Age	Gender	Height Mean (SD)	PEFR value Mean (SD)*
**18-27 Years**	**Male**	171.14 (5.89)	521.54 (55.89)
	**Female**	161.54 (4.73)	417.14 (32.50)
**28-37 Years**	**Male**	174.33 (5.78)	522.17 (58.21)
	**Female**	166.10 (3.69)	445.00 (31.36)
**38-47 Years**	**Male**	172.68 (6.75)	485.35 (52.37)
	**Female**	162.29 (8.72)	422.86 (49.99)
**48-57 Years**	**Male**	172.00 (4.68)	451.47 (42.79)
	**Female**	166.00 (2.28)	413.33 (25.03)
**58-67 Years**	**Male**	169.31 (4.77)	444.38 (36.87)
	**Female**	161.88 (8.61)	398.75 (46.12)

**Figure 1(A) and 1(B):**
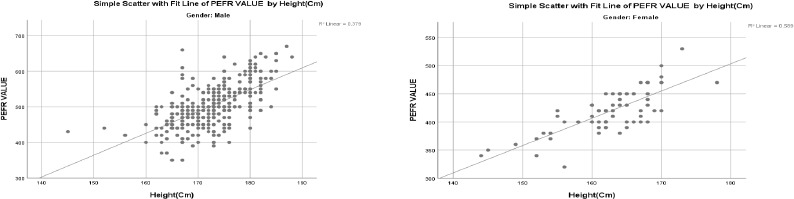
Scatter Graphs of PEFR values to height for males and females

PEFR values were significantly associated with both height and age. Height demonstrated the strongest positive correlation with PEFR, with correlation coefficients of r = 0.616 in males and r = 0.767 in females, indicating that taller individuals generally exhibited higher PEFR values (Figures 1A and 1B). Conversely, age was negatively correlated with PEFR, with coefficients of r = -0.418 in males and r = -0.115 in females, suggesting a decline in lung function with increasing age (Table 2).

**Table 2:** Correlations between PEFR values, age, and height for male and female participants

Gender	Relationships	Pearson correlation	P-VALUE
**Male**	PEFR value and Age	-.418	**.000**
	PEFR value and Height	.616	**.000**
**Female**	PEFR value and Age	-.115	.166
	PEFR value and Height	.767	**.000**

ANOVA results revealed significant effects of both height and age on PEFR for both genders. Height was identified as a dominant predictor of PEFR, with standardized beta coefficients of β = 0.605 (males: p < .001) and β = 0.784 (females: p < .001). This strong positive relationship was supported by high t-values (t = 16.060 for males and t = 10.628 for females, both p < .001), confirming that taller individuals have significantly higher PEFR values.

Age showed a weaker but statistically significant negative relationship with PEFR in both genders. The effect was more pronounced in males (β = -0.402, t = -10.661, p < .001) than in females (β = -0.184, t = -2.502, p = .015), indicating a steeper decline in lung function with age in males.

Overall, multiple linear regression analysis identified height as the strongest positive predictor of PEFR in both males and females, while age contributed to a lesser but still significant decline. Detailed regression results are presented in Tables 3A and 3B.

**Table 3A:** ANOVA Test Results for PEFR (dependent variable) by Gender (Height and Age as predictors)

Gender	Source	Sum of Squares	df	Mean Square	F	Sig.
**Male**	Regression	638508.416	2	319254.208	190.583	**<0.001**
	Residual	542746.630	324	1675.144		
	Total	1181255.046	326			
**Female**	Regression	65852.665	2	32926.333	57.705	**<0.001**
	Residual	39941.855	70	570.598		
	Total	105794.521	72			

Note: Degree of freedom (df).

**Table 3B:** Multiple linear regression analysis for PEFR (dependent variable) with Age and Height as predictor variables

Gender	Model	Beta Value	T Value	P-value
**Male**	Age	-.402	-10.661	**<0.001**
	Height	.605	16.060	**<0.001**
**Female**	Age	-.184	-2.502	**.015**
	Height	.784	10.628	**<0.001**

## Discussion

This study offers important insights into the determinants of Peak Expiratory Flow Rate (PEFR) among healthy Pakistani adults, highlighting the roles of height, age, and gender. The cross-sectional design and consecutive sampling approach allowed for the inclusion of a diverse sample representative of the local adult population attending a tertiary care hospital in Wah Cantt.

Height emerged as the most influential predictor of PEFR. Taller individuals had higher PEFR values, consistent with existing literature suggesting that increased lung volume and thoracic cavity dimensions contribute to stronger expiratory flow (1,17). The anatomical advantage of greater lung surface area allows taller individuals to expel air more forcefully, resulting in higher PEFR measurements. Interestingly, the stronger predictive value of height in females, as shown by the higher beta coefficient, may reflect underlying gender-based differences in airway structure or thoracic anatomy (14).

Age was negatively associated with PEFR in both genders, supporting the well-established pattern of declining pulmonary function over time (18). The more substantial decline observed in males may be attributed to factors such as greater exposure to occupational hazards, differences in lifestyle habits (e.g., physical activity or smoking history), or variations in age-related changes in respiratory muscle strength (5,8). The milder decline in females could potentially be linked to hormonal or physiological factors that help preserve lung function for a longer duration (11).

Gender also significantly influenced PEFR values, with males achieving higher mean PEFR than females, even after controlling for height and age. This is consistent with prior research indicating that males typically possess larger lung volumes and stronger respiratory musculature (1,15). However, the greater impact of height on PEFR among females, as observed in this study, suggests the importance of gender-specific interpretations in clinical assessments (3,14).

These findings emphasize the limitations of generalized PEFR reference ranges and reinforce the need for localized, demographically adjusted norms. The relationships observed between PEFR, and individual characteristics support a tailored approach in respiratory evaluations, allowing for more accurate diagnostics and management of lung function across diverse populations.

While the current study focused on healthy adults, future investigations could extend to include populations with respiratory diseases, the elderly, and individuals with occupational exposures. This would help provide a more comprehensive understanding of PEFR dynamics across different subgroups.

Moreover, longitudinal studies could help track changes in PEFR over time and assess how lifestyle, environmental factors, and healthcare interventions influence pulmonary function (5,16). Collaborations with public health organizations could also facilitate the design and implementation of targeted strategies to improve respiratory outcomes at the community level.

This research contributes significantly to the body of knowledge on respiratory health in Pakistan by establishing baseline PEFR profiles for healthy adults in Wah Cantt. The findings can inform both clinical practice and public health planning, offering healthcare providers a data-driven basis for tailoring respiratory care interventions.

In conclusion, this study confirms a strong positive correlation between height and PEFR among healthy Pakistani adults, establishing height as a key predictor of lung function across genders. A negative correlation with age was also observed, more pronounced in males, consistent with known age-related physiological declines. Males exhibited higher PEFR values overall, reflecting gender-based anatomical and physiological differences. Generally, PEFR demonstrates a strong positive correlation with height and is generally higher in males compared to females. Additionally, PEFR declines with increasing age, with the rate of decline being more pronounced in males.

These findings underscore the importance of incorporating demographic factors—particularly height, age, and gender—into the interpretation of PEFR values. Personalized lung function assessment enhances diagnostic precision and ensures more effective and equitable respiratory care. Future research should investigate additional influences such as environmental exposure, nutrition, and physical activity to develop more robust predictive models and improve clinical outcomes in respiratory health.
